# Inhibition of Uncoupling Protein 2 Enhances the Radiosensitivity of Cervical Cancer Cells by Promoting the Production of Reactive Oxygen Species

**DOI:** 10.1155/2020/5135893

**Published:** 2020-03-04

**Authors:** Cui Hua Liu, Zhe Hao Huang, Xin Yu Dong, Xin Qiang Zhang, Yuan Hang Li, Gang Zhao, Bao Sheng Sun, Yan Nan Shen

**Affiliations:** ^1^NHC Key Laboratory of Radiobiology, School of Public Health, Jilin University, Changchun 130021, China; ^2^Department of Neurosurgery, China-Japan Union Hospital of Jilin University, Changchun 130031, China; ^3^Department of Radiotherapy, Tumor Hospital of Jilin Province, Changchun 130012, China

## Abstract

**Objective:**

The mechanism of enhanced radiosensitivity induced by mitochondrial uncoupling protein UCP2 was investigated in HeLa cells to provide a theoretical basis as a novel target for cervical cancer treatment.

**Methods:**

HeLa cells were irradiated with 4 Gy X-radiation at 1.0 Gy/min. The expression of UCP2 mRNA and protein was assayed by real-time quantitative polymerase chain reaction and western blotting. UCP2 siRNA and negative control siRNA fragments were constructed and transfected into HeLa cells 24 h after irradiation. The effect of UCP2 silencing and irradiation on HeLa cells was determined by colony formation, CCK-8 cell viability, *γ*H2AX immunofluorescence assay of DNA damage, Annexin V-FITC/PI apoptosis assay, and propidium iodide cell cycle assay. The effects on mitochondrial structure and function were investigated with fluorescent probes including dichlorodihydrofluorescein diacetate (DCFH-DA) assay of reactive oxygen species (ROS), rhodamine 123, and MitoTracker Green assay of mitochondrial structure and function.

**Results:**

Irradiation upregulated UCP2 expression, and UCP2 knockdown decreased the survival of irradiated HeLa cells. UCP2 silencing sensitized HeLa cells to irradiation-induced DNA damage and led to increased apoptosis, cell cycle arrest in G2/M, and increased mitochondrial ROS. Increased radiosensitivity was associated with an activation of P53, decreased Bcl-2, Bcl-xl, cyclin B, CDC2, Ku70, and Rad51 expression, and increased Apaf-1, cytochrome c, caspase-3, and caspase-9 expression.

**Conclusions:**

UCP2 inhibition augmented the radiosensitivity of cervical cancer cells, and it may be a potential target of radiotherapy of advanced cervical cancer.

## 1. Introduction

Cervical cancer is a significant threat to women's health. Vaccination or screening for early diagnosis has reduced the incidence of cervical cancer in developed countries, but it is the fourth highest cause of cancer-related death in the developing world [1]. Radiotherapy is frequently used to treat terminal cervical cancer, especially in poor countries, but its effect on prognosis is limited by a reduction of radiosensitivity and lack of understanding of the molecular mechanisms underlying radiosensitivity [2–4]. It would be helpful to find molecular targets that are radiosensitive.

Mitochondrial uncoupling proteins (UCPs) inhibit oxidative phosphorylation and regulate the generation of mitochondrial reactive oxygen species (ROS) including hydroxyl and hydroperoxyl radicals, hydrogen peroxide, and superoxides [5, 6]. Inhibition of UCP activity increases the formation of ROS, mitochondrial transition pore permeability, and damage to DNA, proteins, and lipids that causes changes in cell growth [7, 8]. Five UCPs have been identified in mammals. UCP1 is primarily expressed in brown adipose tissue. UCP2 is expressed in the liver, brain, pancreas, adipose, spleen, kidney tissue, and cells in the central nervous and immune systems [9–14]. UCP4 and UCP5/BMCP1 are expressed in a tissue-specific manner and act to reduce mitochondrial membrane potential [15]. The physiological and pathological activities of UCPs include involvement in neurodegenerative diseases, atherosclerosis, and various human cancers. UCP2 is overexpressed in various cancers. Overexpression is associated with downregulation of ROS, which provides a growth advantage for cancer cells, and autophagy; both of which are altered in cancer cells [16–19]. UCP2 expression is increased in most human colon cancers, and the expression level was found to be correlated with the degree of neoplastic change [20]. Previous studies have found significant associations between mitochondrial UCP2 and tumor grade in primary breast cancer and reduced sensitivity of breast cancer cells to therapeutic agents by increasing oxidative stress, and some studies show that UCP2 has a critical role in chemotherapeutic drug resistance in pancreatic ductal adenocarcincoma [21–23]. Few data are available on the role of UCP2 in cervical cancer. This study found a significant elevation of UCP2 expression and ROS production in irradiated HeLa cervical cancer cells that inhibited cell proliferation and promoted apoptosis. UCP2 may be a target molecule for the development of novel cervical cancer-specific radiosensitizers.

## 2. Materials and Methods

### 2.1. Cell Culture, Transfection, and Irradiation

HeLa cells were obtained from national experimental cell resource sharing platform and plated and cultured in minimum essential medium (MEM) plus 10% fetal bovine serum and 1% penicillin and streptomycin (Gibco, Grand Island, NY, USA) at 37°C and 5% CO_2_. The small interfering (si)RNA specific for UCP2 or an siRNA negative control (siNC) was transfected with Rfect transfection regent (Shanghai YESEN Biotechnology Co., Ltd.). UCP2 mRNA and protein expression were determined by quantitative real-time polymerase chain reaction (qPCR) and western blotting. Cells were exposed to 4 Gy (1.02 Gy/min at180 kV at a distance of 0.7 m using an X-RAD320 X-ray irradiator (Precision X-ray, North Branford, CT, USA).

### 2.2. qPCR

RNA was extracted from cells at 0, 3, 6, 12, and 24 h after irradiation using TRIzol reagent (Invitrogen, Carlsbad, CA, USA), and complementary DNA was synthesized with a reverse transcription kit (Takara Bio Inc., Japan) following the manufacturer's instructions. The qPCR reaction conditions were 94°C denaturation for 30 s, 57°C annealing for 30s, 72°C extension for 30 s, and for 33 cycles of augmentation. *β*-Actin was the negative control. The primer sequences were *β*-actin forward, 5′-TGACGTGGACATCCGCAA-AG-3′; reverse 5′-CTGGAAGGTGGACAGCGA-GG-3′; UCP2, forward 5′-CCCCGAAGCCTCTACAAT-GG-3′, reverse 5′-CTGAGCTTGGAATCGGAC-CTT-3′. There were 3 replicate wells per group, and the experiment was performed in triplicate.

### 2.3. Clonogenic and Cell Proliferation Assays

Six-well plates were seeded with 2 × 103 transfected cells per well, treated with 0, 1, 2, 3, 4, or 5 Gy X-irradiation after 24 h and then cultured for an additional 14 days. The resulting colonies were washed twice with phosphate buffered saline (PBS), fixed with 4% paraformaldehyde for 10 min and then stained with 0.5% crystal violet for 30 min. After washing with deionized water, the plates were air dried at room temperature to count the number of colonies. The maximum inhibition was observed with 4 Gy, which was used for the cell proliferation assay. There were 3 replicate wells per group, and the experiment was performed in triplicate. Proliferation was assayed 24 h after exposure to X-irradiation using a cell-counting kit (CCK)-8 following the manufacturer's instructions. After adding 10 *μ*l CCK-8 to each well, the plates were incubated in the dark at 37°C for 3 h. Absorbance was measured with a microplate reader. There were 5 replicate wells per group, and the experiment was performed in triplicate.

### 2.4. Flow Cytometry Cell Cycle, Apoptosis, and Autophagy Assays

Harvested cells were fixed for 2 h in 150 *μ*l PBS and 400 *μ*l 100% ethanol at 4°C, centrifuged, and washed twice. Cells were suspended in propidium iodide (PI) staining solution (1× PBS, 250× PI stock solution, 1000× RNase), incubated in the dark for 30 min at room temperature, and transferred to tubes for analysis with a FACSCalibur flow cytometer (BD Biosciences, Franklin Lakes, NJ). Apoptosis was assayed with an Annexin V-FITC apoptosis detection kit (BD Biosciences). HeLa cells were washed twice in PBS and resuspended in binding buffer (2 × 10^5^ cells/ml). A total volume of 195 *μ*l of the cell suspension was incubated with 5 *μ*l Annexin V-FITC for 10 min at room temperature. After washing with PBS, the cells were costained with PI and assayed. For the autophagy assay, cells were seeded into 6-well plates and cultured overnight before study treatment for an additional 24 h. After treatment, cells were washed twice with cold PBS. Autophagic vacuoles were labeled by incubating cells in MEM containing 50 mM monodansylcadaverine (MDC)/well at 37°C for 1 h. Cells then suspended, were washed with PBS, and fixed in 4% paraformaldehyde for 15 min. Fluorescence was read by flow cytometry. The experiment was performed in triplicate.

### 2.5. *γ*H2AX Assay

Phosphorylation of the Ser-139 residue of the histone H2AX forms *γ*H2AX, a marker of DNA damage. After treatment, the cells were washed twice with PBS, fixed with 4% paraformaldehyde for 20 min, and permeabilized with 0.1% NP-40 in PBS for 15 min at room temperature. After blocking in PBS with 0.1% NP-40 and 10% bovine serum albumen for 30 min, the cells were incubated with anti-*γ*H2AX antibody (Cell Signaling Technology, Danvers, MA, USA) 1 : 500 dil in blocking buffer for 24 h at 4°C and then incubated with an Alexa fluor 488-conjugated secondary antibody (1 : 500 in blocking buffer) for 1 h at room temperature. The nuclei were counterstained with 4′,6-diamidino-2-phenylindole (DAPI) 100 ng/ml for 5 min. The samples were mounted and observed with a laser scanning microscope (LSM 510; Carl Zeiss, Oberkochen, Germany). Cells with ten or more foci were considered positive for damaged and unrepaired DNA. The experiment was performed in triplicate.

### 2.6. Mitochondrial Membrane Potential and Integrity

The fluorescence intensity of rhodamine 123 (Rh123, Sigma-Aldrich, St. Louis, MO, USA) is inversely proportional to the mitochondrial membrane potential. After 24 h of incubation in 24-well plates (8 × 10^4^ cells/well), transfected and nontransfected cells were exposed to 4 Gy X-irradiation. The cells were harvested by trypsin digestion and incubated in PBS containing 5 *μ*M rhodamine 123 for 30 min at 37°C. Fluorescence intensity was measured by flow cytometry. MitoTracker Green FM (Yeasen Biotechnology, Shanghai, China) is a fluorescent probe that binds to mitochondrial lipids regardless of the mitochondrial membrane potential and does not fluoresce in aqueous solution. It is used to indicate the integrity of mitochondrial membranes. After 24 h of incubation, transfected and nontransfected cells were plated on cover slips (1.2 × 10^5^ cells/slip), and cells were irradiated with 4 Gy X-irradiation. After irradiation, cells were incubated in PBS with 200 nM MitoTracker Green FM for 20 min at 37°C, washed twice with PBS, and then observed with a fluorescence microscope (Leica). The experiment was performed in triplicate.

### 2.7. ROS Production

2,7-Dichlorodihydroflurescein diacetate (DCFH-DA, Sigma-Aldrich) is oxidized to highly fluorescent dichlorofluorescein (DCF) by ROS and was used to assay intracellular ROS production. After 24 h of incubation, transfected and nontransfected cells were plated in 96-well plates (6 × 10^3^ cells/well) and exposed to 4 Gy X-irradiation. Six, 12, and 24 h after irradiation, cells were incubated in PBS containing 8 *μ*M DCF-DA for 15 min at 37°C and were washed twice with PBS, and then the fluorescence was read by a Cytation-3 Cell Imaging Multi-ModeReader System (BioTek, Winooski, Vermont, USA). There were 5 replicate wells per assay, and the assays were performed in triplicate.

### 2.8. Western Blotting

Total protein was extracted from cells at 0, 3, 6, 12, and 24 h after irradiation in pH 7.5 RIPA lysis and extraction buffer (50 mM Tris–HCl, 150 mM NaCl, 1% NP-40, 50 mM NaF, 200 *μ*M Na_3_PO_4_, 1 mM EDTA, 0.5% sodium deoxycholate, 0.1% sodium dodecyl sulfate) and protease inhibitor cocktail (Roche, Meylan, France). After the total proteins were quantitatively determined, 30 *μ*g proteins were separated by 15% sodium dodecyl sulfate-polyacrylamide gel electrophoresis (SDS-PAGE, 90 V, 90 min) and then blotted onto nitrocellulose membranes (Merck Millipore, Billerica, MA, USA). The membranes were blocked at room temperature for 1 h in TBST containing 5% nonfat milk and then incubated with anti-Bcl-2, anti-Bcl-xl, anti-caspase 3, anti-caspase 9, anti-cyclin B (Cell Signaling Technology, Danvers, MA, USA), anti-cytochrome c, anti-Ku70, anti-Rad51 (ImmunoWay, Plano, TX, USA), anti-Apaf-1, anti-CDC2, anti-P53, and anti-*β*-actin (Bioworld Technology Inc., USA), 1 : 1000 dil overnight at 4°C. After washing with TBST, the membranes were incubated with secondary antibodies (ImmunoWay, Plano, TX, USA) at 1 : 10000 dil for 1 h at room temperature. Proteins were visualized with an enhanced chemiluminescence kit (Santa Cruz Inc., Santa Cruz, CA, USA). The bands were scanned for grayscale ratio analysis. The experiment was performed in triplicate.

### 2.9. Statistical Analysis

Statistical analysis was performed with SPSS, version 24.0 (SPSS, IBM Corp, Armonk, NY, USA). The results were presented as mean ± SD and subjected to one-way ANOVA; *P* < 0.05 was considered as significant.

## 3. Results

### 3.1. Induction of UCP2 Expression in Cervical Cancer Cells by Irradiation

**Figure 1:**
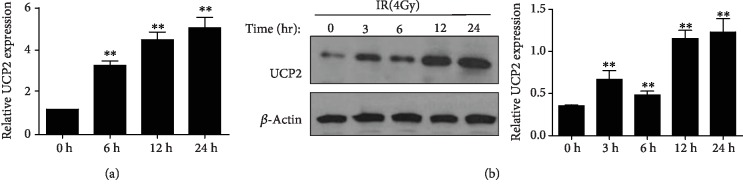
UCP2 expression was upregulated in response to ionizing radiation in HeLa cells. (a) UCP2 mRNA level at 6 h, 12 h, and 24 h after irradiation was determined with qRT-PCR. (b) UCP2 protein level at 3 h, 6 h, 12 h, and 24 h after irradiation was determined by western blotting. Bars are means ± SD of triplicate assays. ∗∗*P* < 0.01 vs. 0 h X-radiation exposure.

UCP2 expression in HeLa cells was assayed at different times after irradiation, and the qPCR and western blot results indicate that both UCP2 mRNA and protein levels increased in response to irradiation (Figure 1).

### 3.2. Inhibition of UCP2 Induction Sensitizes HeLa Cells to Irradiation

**Figure 2:**
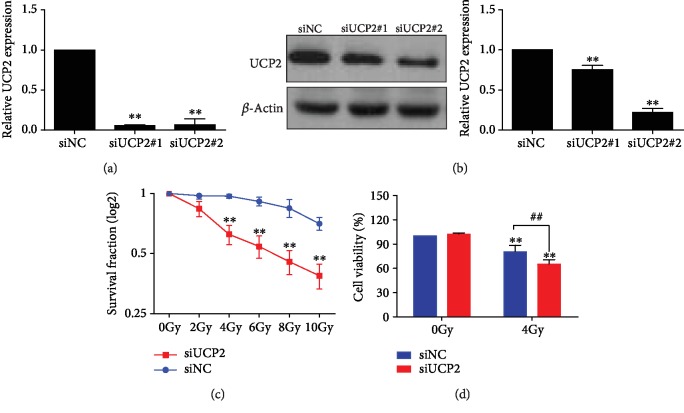
UCP2 knockdown enhanced the radiosensitivity of HeLa cells. (a) Relative amount of UCP2 mRNA after transfection with UCP2 siRNA or siNC. (b) The amount of UCP2 protein after transfection with UCP2 siRNA or siNC. (c, d) Cell proliferation and survival were determined by colony formation and CCK-8 assays. Bars are means ± SD of triplicate assays. ∗*P* < 0.05 vs. controls; #*P* < 0.05 vs. 4 Gy irradiation.

The impact of UCP2 on the response to irradiation was investigated in HeLa cells transfected with UCP2 siRNA#1 and UCP2 siRNA#2. Both siRNAs significantly suppressed UCP2 protein (Figure 2(b)) and mRNA levels (Figure 2(a)). UCP2 siRNA#2 was chosen for use in subsequent procedures. UCP2 knockdown decreased the survival of HeLa cells following irradiation. Both cell proliferation assayed by colony formation (Figure 2(c)) and cell viability assayed by the CCK-8 kit (Figure 2(d)) were decreased compared with control cells. The results indicate that the silencing of UCP2 expression enhanced the radiation sensitivity of HeLa cells.

### 3.3. Silencing UCP2 Promotes Radiation-Induced Apoptosis of HeLa Cells

**Figure 3:**
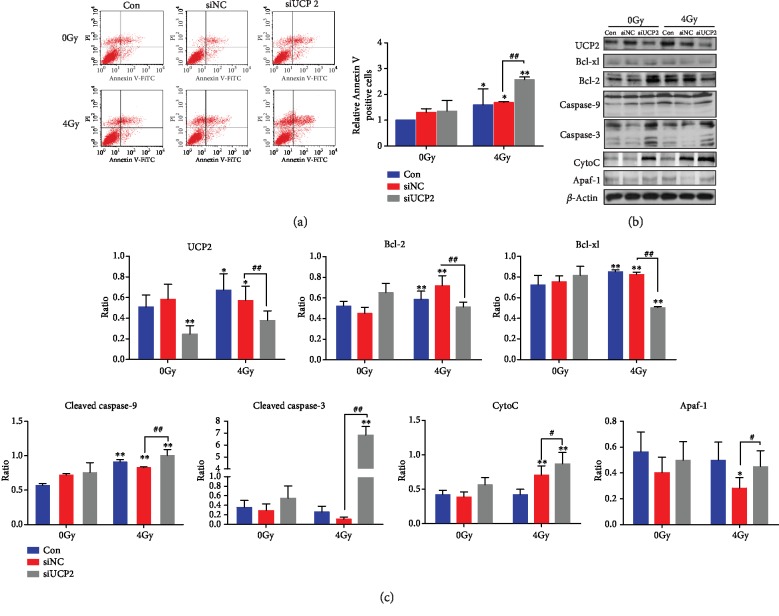
UCP2 knockdown increases apoptosis of irradiated HeLa cells. (a) Flow cytometry analysis of apoptosis after UCP2 knockdown with or without irradiation. (b) Western blots of Bcl-2, Bcl-xl, caspase-3, caspase-9, cytochrome c, Apaf-1, and the *β*-actin protein loading control. (c) Relative protein amount of Bcl-2, Bcl-xl, caspase-3, caspase-9, cytochrome c, and Apaf-1. Bars are means ± SD of triplicate assays. ∗*P* < 0.05 and ∗∗*P* < 0.01 vs. control, #*P* < 0.05 and ##*P* < 0.01 vs. 4 Gy irradiation.

The effect of UCP2 on HeLa cell apoptosis was assayed by flow cytometry. Exposure to ionizing radiation led to increased apoptosis, and the increase was greater in cells with UCP2 knockdown than in the negative control cells. UCP2 depletion did not significantly alter apoptosis in the absence of irradiation. As shown in Figure 3(a), the increase in Annexin-V-positive cells following irradiation was greater in UCP2 knockdown than in control cells. The level of apoptosis proteins was changed by UCP2 knockdown. The level of Bcl-xl, Bcl-2, caspase-9, caspase-3, cytochrome c, and Apaf-1 in control and in siNC and siUCP2 transfected HeLa cells with and without irradiation is shown in Figures 3(b) and 3(c). Irradiation significantly increased caspase-9, caspase-3, cytochrome c, and Apaf-1 levels and decreased Bcl-xl and Bcl-2 amounts in siUPC2-transfected cells. Overall, the results indicate that UCP2 knockdown increased the radiosensitivity of HeLa cells at least in part by activating mitochondria-dependent apoptosis.

### 3.4. Downregulation of UCP2 Induces Cell Cycle Arrest of Irradiated HeLa Cells

**Figure 4:**
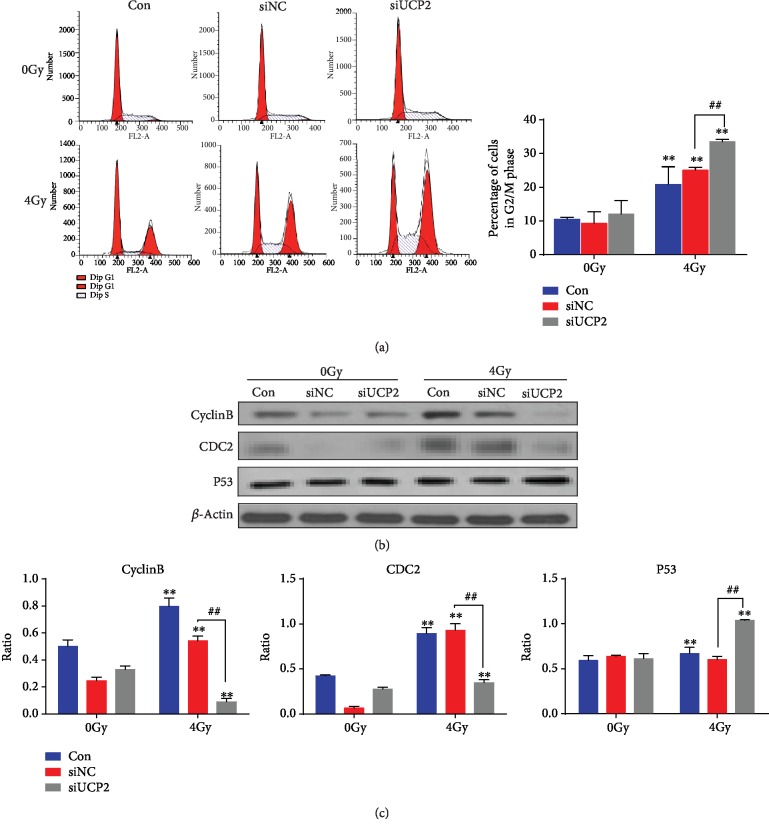
UCP2 knockdown and cell cycle arrest of irradiated cancer cells in G2/M. (a) Flow cytometry of HeLa cells after UCP2 knockdown with or without irradiation. (b) Western blots of cyclin B, CDC2, P53, and the *β*-actin loading control expression. (c) Relative protein amount of cyclin B, CDC2, and P53. Bars and data are means ± SD of triplicate assays. ∗*P* < 0.05 and ∗∗*P* < 0.01 versus control, #*P* < 0.05 and ##*P* < 0.01 vs. 4 Gy irradiation.

Knockdown of UCP2 resulted in an increased accumulation of HeLa cells in the G2/M phase of the cell cycle after 24 h compared with control cells. Irradiation of UCP2 knockdown cells resulted in a more pronounced G2/M block after 24 hours (Figure 4(a)). The effects of UCP2 knockdown on the amount of proteins that regulate the G2/M phase are shown in Figures 4(b) and 4(c). Radiation and siUCP2 significantly increased p53 level and decreased the amount of cyclin B and CDC2. Silencing UCP2 increased the G2/M arrest of HeLa cells following radiation treatment.

### 3.5. Knockdown of UCP2 Reduced the Ability of DNA Damage Repair

**Figure 5:**
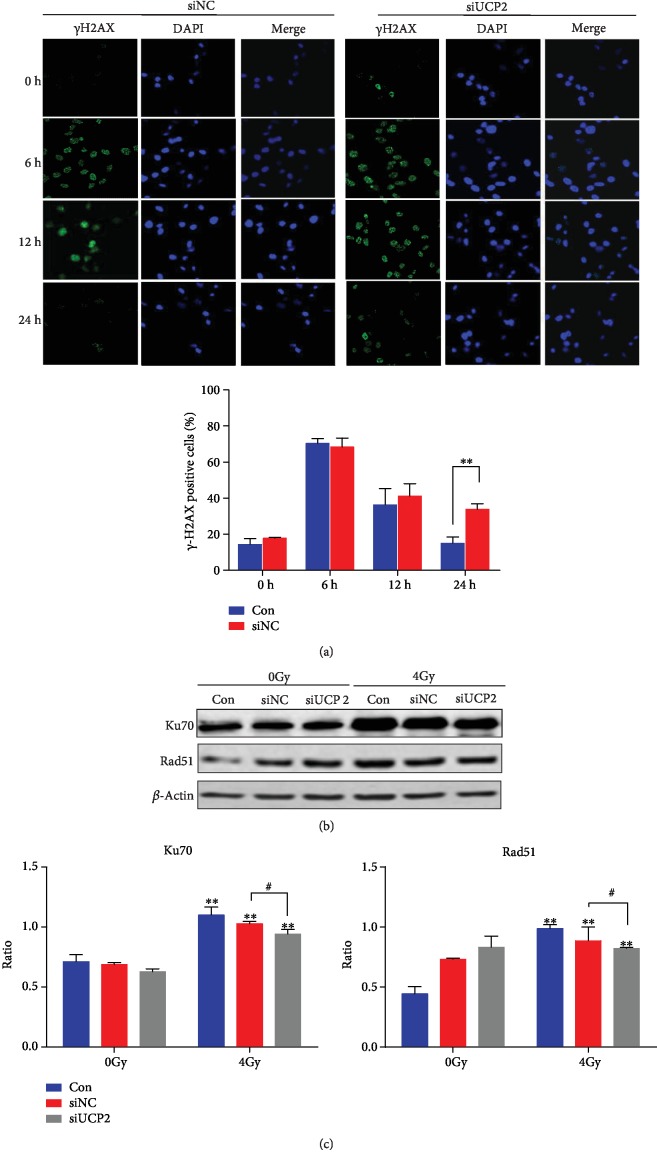
Effect of UCP2 knockdown on DNA damage repair in HeLa cells. (a) *γ*H2AX expression assayed by immunofluorescence. (b) Western blots of Ku70, Rad51, and the *β*-actin loading control. (c) Relative protein amount of Ku70 and Rad51. Bars represent means ± SD of triplicate assays. ∗∗*P* < 0.01 vs. negative control, #*P* < 0.05 and ##*P* < 0.01 vs. 4 Gy irradiation.

Ionizing radiation produces DNA double-strand breaks, and the initial response to DNA damage is phosphorylation of H2AX [24]. The effect of UCP2 knockdown on the repair of DNA damage in cervical cancer cells was investigated by assaying *γ*H2AX protein level at different times after irradiation. As shown in Figure 5(a), 16.3% of nonirradiated HeLa cells transfected with siNC and 18.5% transfected with siUCP2 group were *γ*H2AX positive at 6 h. At 12 h, the corresponding percentages were 37.5% and 40.2%. The between-group differences were not significant. The extent of DNA repair is associated with a decrease in the expression of *γ*H2AX at 24 h. The percentage of *γ*H2AX-positive cells was significantly higher in siUCP2 than in siNC cells (29.8% vs. 10.0%, *P* < 0.05). The level of proteins of Ku70 and Rad51, which are proteins associated with DNA repair, was higher in irradiated than in nonirradiated cells, and following irradiation was lower in UCP2 knockdown than in siNC cells (Figures 5(b) and 5(c)). The results show that UCP2 expression delayed the repair of radiation-induced DNA damage.

### 3.6. Silencing UCP2 Increases Intracellular ROS Production after Irradiation

**Figure 6:**
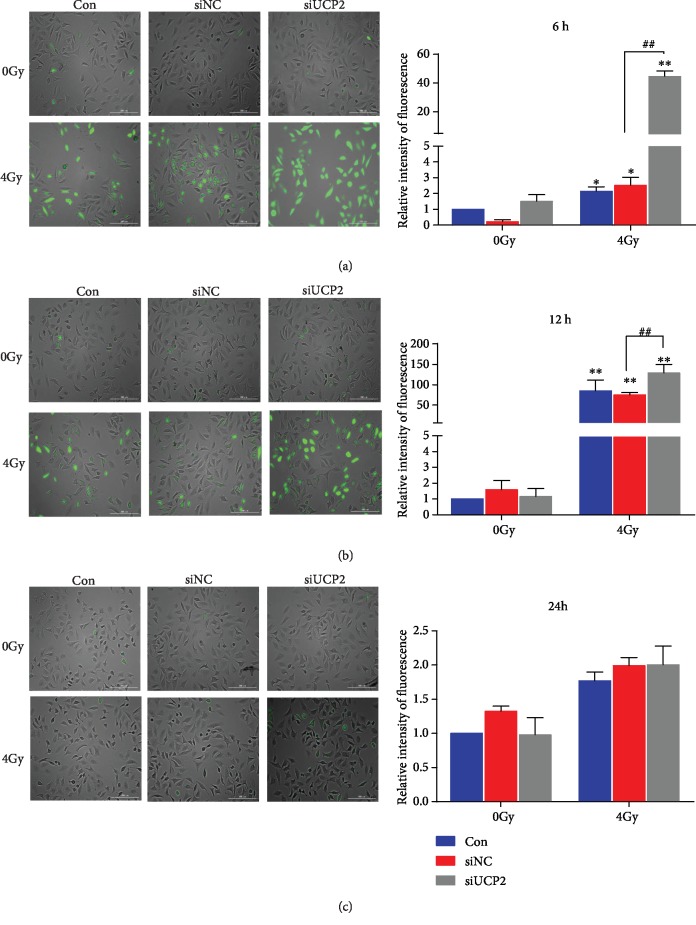
Change in intracellular ROS at (a) at 6 h (b) 12 h, and (c) 24 h after irradiation. Bars represent means±SD of triplicate assays. ∗∗*P* < 0.01 vs. control; ##*P* < 0.01 vs. 4 Gy irradiation.

Ionizing radiation promotes ROS production in mitochondria leading to changes in structure and function [25]. As shown in Figures 6(a)–6(c), ROS production was lower in unirradiated than in irradiated cells at 6 and 12 h, and UCP2 knockdown enhanced production of ROS in irradiated cells. There was no significant difference in ROS production among the three groups at 24 h. Probably because of an early event in ROS production, UCP2 knockdown promoted an increase in membrane potential, and as ROS production increased, it promoted an increase of membrane permeability that led to the collapse of membrane potential and the dissipation of ROS.

### 3.7. UCP2 Inhibition Alters the Mitochondrial Membrane Potential of Irradiated HeLa Cells

**Figure 7:**
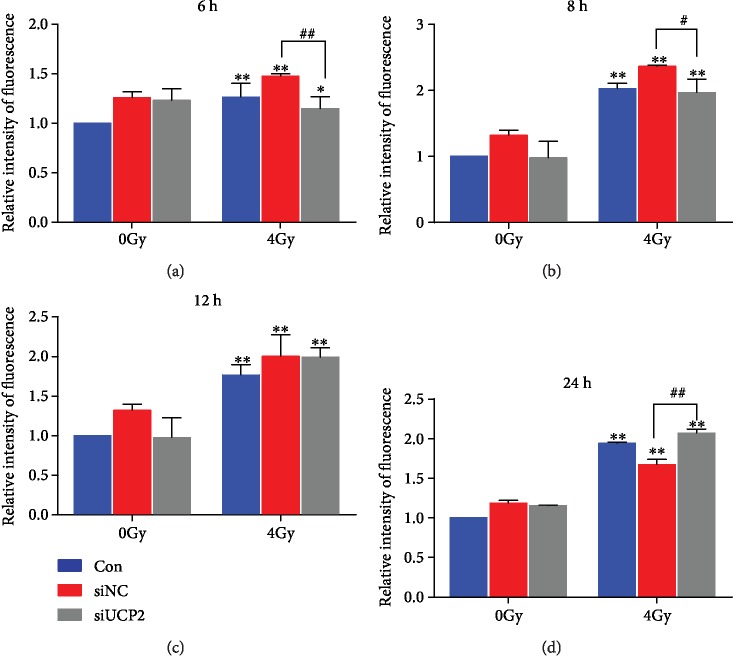
Change in mitochondrial membrane potential after irradiation in the three study groups. Bars represent means ± SD of triplicate assays. ∗*P* < 0.05 and ∗∗*P* < 0.01 vs. control; #*P* < 0.05 and ##*P* < 0.01 vs. 4 Gy irradiation.

UCP2 mediates proton leakage that lowers membrane potential and is reversed when its expression is decreased [26]. Mitochondrial membrane potential was significantly lower in irradiated than in unirradiated cells over the 24 h of measurement. At 6 and 8 h after irradiation, the mitochondrial membrane potential was higher in siUCP2 than in other cells (Figures 7(a) and 7(b)). If UCP2 silencing inhibits proton leakage, then the increase in membrane potential will promote ROS production. At 12 h after irradiation, there were no significant differences among the three groups (Figure 7(c)), but at 24 h, the mitochondrial membrane potential was lower in the siUCP2 than in the other study groups (Figure 7(d)). The results indicate that ROS enhanced the permeability of the mitochondrial inner membrane to protons resulting in the decrease or collapse of membrane potential. The changes in mitochondrial membrane potential are shown in [Supplementary-material supplementary-material-1]. The results are consistent with the changes of the mitochondrial membrane potential in siUCP2 cells following irradiation that affected normal physiological function.

### 3.8. UCP2 Silencing Induces Loss of Mitochondrial Function

**Figure 8:**
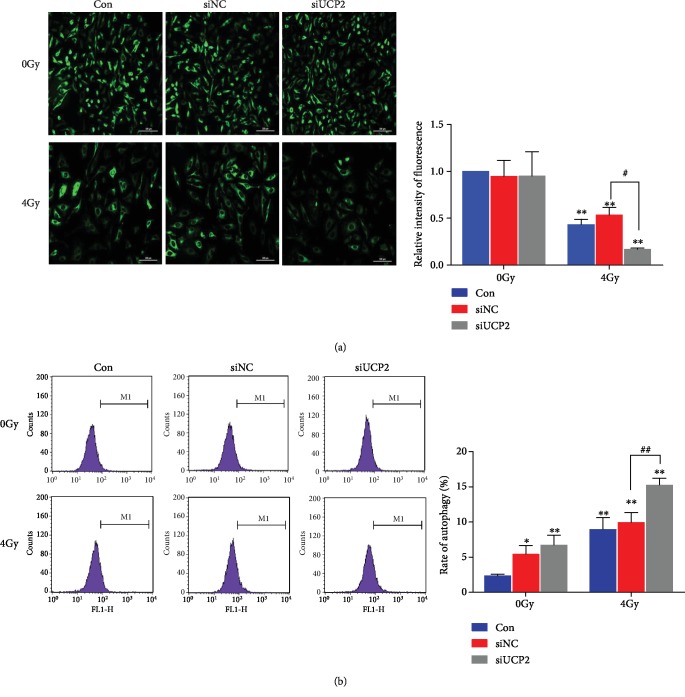
UCP2 knockdown destroyed mitochondria and enhanced autophagy of HeLa cells. (a) Radiation-induced disruption of mitochondrial membrane integrity. (b) UCP2 knockdown enhanced radiation-induced autophagy. Bars are means ± SD of triplicate assays. ∗*P* < 0.05 and ∗∗*P* < 0.01 vs. controls; #*P* < 0.05 and ##*P* < 0.01 vs. 4 Gy irradiation.

Oxidation of the mitochondrial matrix increases the permeability of the inner membrane, loss of membrane integrity, and mitochondrial rupture [8]. HeLa cells transfected with siNC or siUCP2 were either irradiated with 4 Gy or not treated and were probed with MitoTracker Green after 24 h (Figure 8(a)). In unirradiated cells, the mitochondrial membrane was intact. After irradiation, the integrity of the mitochondrial membrane was reduced, most obviously in UCP2 knockdown cells. The results indicate that UCP2 knockdown increased the severity of the mitochondrial membrane damage caused by irradiation. Transfection of siNC and siUCP2 increased autophagy in unirradiated cells, which may have been caused by toxicity of transfection reagents and siRNA, and irradiation significantly increased autophagy after UCP2 silencing (Figure 8(b)). Autophagy was enhanced after the destruction of mitochondria, which further promoted radiation-induced cell death.

## 4. Discussion

Cervical cancer is one of the most common malignancies and the fourth leading cause of cancer death in women worldwide [27]. Surgery and radiotherapy supplemented by chemotherapy are standard treatments for early-stage disease. More advanced cervical cancer is treated mainly by concurrent radiotherapy and chemotherapy [28, 29]. Cervical cancer is radiosensitive, but radiotherapy resistance has been reported in both cervical cancer cells and clinical practice. The resistance in radiotherapy is a frequent cause of treatment failure [30]. The causes of radiotherapy resistance are not completely clear but include hypoxia. Mitochondria reduce ROS production to promote cancer cell survival through a complex regulatory feedback mechanism that may involve UCP2 [31]. UCP2 involvement has been reported in many diseases. For example, increased expression of UCP2 can suppress glucose-stimulated insulin secretion, and inhibition of UCP2 expression was shown to decrease the growth of PaCa44 human pancreatic cancer cells [32, 33]. UCP2 can regulate the proliferation and differentiation of neural stem cells by inhibiting ROS production, and UCP2 overexpression can decrease basal autophagy [34–36]. An increase in the radiosensitivity of cervical cancer cells caused by UCP2 has not been previously reported.

The study results suggest that the genes coding UCP2 may be radiation induced, with similar background expression in different types of cervical cancer cells. UCP2 silencing increased the radiosensitivity of HeLa cells by inhibiting colony formation and cell proliferation. UCP2 silencing-induced radiosensitization was associated with increased ROS production that led to apoptosis and impaired autophagy. Apoptosis of HeLa cells was significantly increased by combining UPC2 silencing and irradiation compared with either treatment alone. UCP2 silencing may have increased radiosensitivity by enhancing apoptosis, and the results of the MDC assay found that UCP2 silencing may blocked the normal process of autophagy.

In addition to aerobic metabolism and energy production, mitochondria can induce apoptosis and control the survival and death of eukaryotic cells [37, 38]. Stability of the mitochondrial transmembrane potential is important for the maintenance of normal cellular physiology [39]. Changes of mitochondrial membrane permeability, decrease in the transmembrane potential, and activation of Bcl-2 family and caspase proteins ultimately lead to cell apoptosis [40]. In this study, inhibition of UCP2 expression in HeLa cells combined with ionizing radiation reduced the mitochondrial transmembrane potential, consistent with the damage of the inner membrane and resulting in increased apoptosis and inhibition of autophagy. ROS are signal molecules that regulate cellular physiology, but excess ROS production causes oxidative damage to cellular membranes and molecules [41, 42]. Mitochondria are both sites of ROS production and ROS targets. In this study, MitoTracker Green staining found that irradiation of HeLa cells transfected with siRNA to inhibit UCP2 expression resulted in damage to mitochondrial membranes.

**Figure 9:**
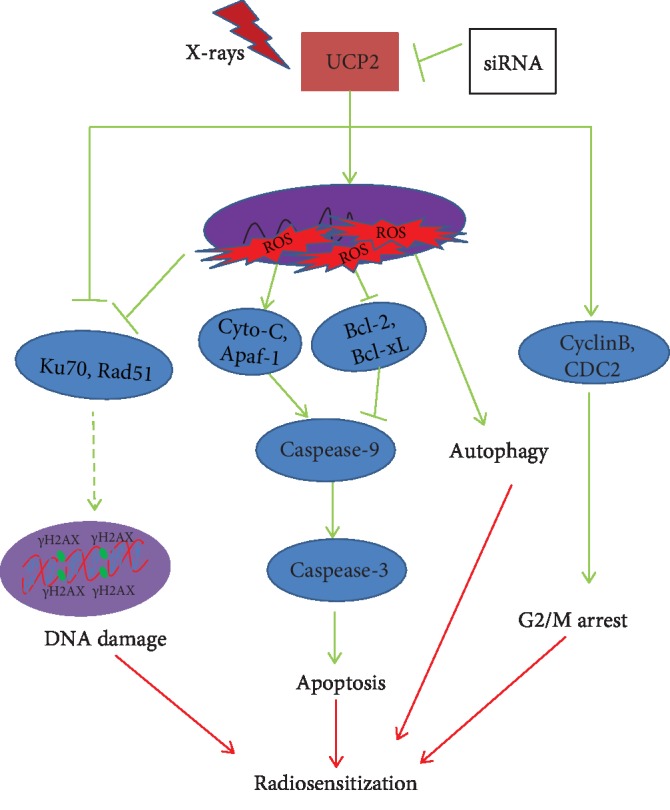
Potential mechanism of UCP2 regulation of the radiosensitivity of HeLa human cervical cancer cells.

UCP2 silencing and irradiation resulted in an increase in the percentage of cells in the G2/M phase of the cell cycle and *γ*H2AX foci assays showed that UCP2 silencing increased radiation-induced DNA damage. Western blot assays revealed that UCP2 silencing inhibited the expression of Bcl-2, an antiapoptosis protein, Ku-70, and Rad51, which are active in DNA repair, cyclin B, and CDC2, which regulate the G2/M phase checkpoint. UCP2 silencing increased the expression of cytochrome c, Apaf-1, caspase-3, and caspase-9, which are proapoptotic proteins, and P53, which induces DNA damage in HeLa cells. The protein expression results confirmed that UCP2 silencing contributed to overcoming the radioresistance of cervical cancer cells by apoptosis, G2/M arrest, and DNA damage. The overall results support the potential utility of UCP2 as an antitumor target in cervical cancers and as a radiosensitizer for HeLa cells (Figure 9).

In summary, this is the first report of the reversal of radioresistance in cervical cancer cells caused by loss of UPC2 expression. UPC2 might be a useful target for the development of novel radiosensitizers. Further experimental and clinical studies are needed to reveal the function of UCP2 in radiosensitivity.

## Data Availability

The data that support the findings of this study are available from the corresponding author upon reasonable request.

## Supplementary Materials

Supplementary Materials Flow diagram of alteration of mitochondrial membrane potential (A, B, C, D). Alteration of mitochondrial membrane potential after irradiation in 6 h, 8 h, 12 h, and 24 h.
