# Corrigendum to “Inhibition of Uncoupling Protein 2 Enhances the Radiosensitivity of Cervical Cancer Cells by Promoting the Production of Reactive Oxygen Species”

**DOI:** 10.1155/2021/1841783

**Published:** 2021-01-11

**Authors:** Cui Hua Liu, Zhe Hao Huang, Xin Yu Dong, Xin Qiang Zhang, Yuan Hang Li, Gang Zhao, Bao Sheng Sun, Yan Nan Shen

**Affiliations:** ^1^NHC Key Laboratory of Radiobiology, School of Public Health, Jilin University, Changchun 130021, China; ^2^Department of Neurosurgery, China-Japan Union Hospital of Jilin University, Changchun 130031, China; ^3^Department of Radiotherapy, Tumor Hospital of Jilin Province, Changchun 130012, China

In the article titled “Inhibition of uncoupling protein 2 enhances the radiosensitivity of cervical cancer cells by promoting the production of reactive oxygen species” [1], an error was identified in Figure 3 which was introduced by the authors during figure preparation. Figure 3(a) is therefore corrected as below:

## Figures and Tables

**Figure 1:**
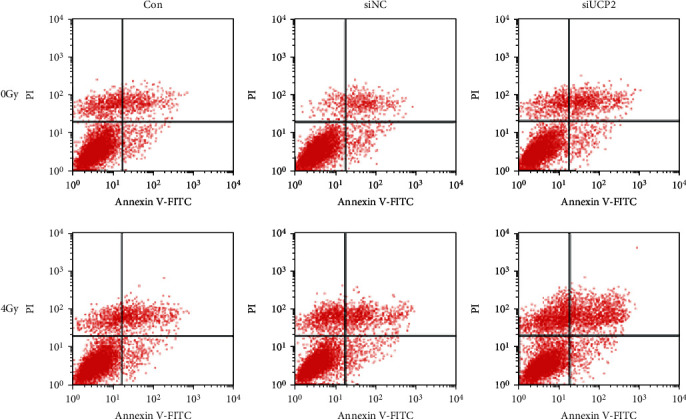
UCP2 knockdown increases apoptosis of irradiated HeLa cells. (a) Flow cytometry analysis of apoptosis after UCP2 knockdown with or without irradiation.

## References

[1] Liu C. H., Huang Z. H., Dong X. Y. (2020). Inhibition of uncoupling protein 2 enhances the radiosensitivity of cervical cancer cells by promoting the production of reactive oxygen species. *Oxidative Medicine and Cellular Longevity*.

