# Supplementary material for: Inhibition of Uncoupling Protein 2 Enhances the Radiosensitivity of Cervical Cancer Cells by Promoting the Production of Reactive Oxygen Species
Source: Oxid Med Cell Longev. 2020 Mar 4;2020:5135893. doi: 10.1155/2020/5135893 (PMC7073473; doi:10.1155/2020/5135893)
Supplement: Supplementary Materials — Flow diagram of alteration of mitochondrial membrane potential (A, B, C, D). Alteration of mitochondrial membrane potential after irradiation in 6 h, 8 h, 12 h, and 24 h. [file 5135893.f1.pdf]

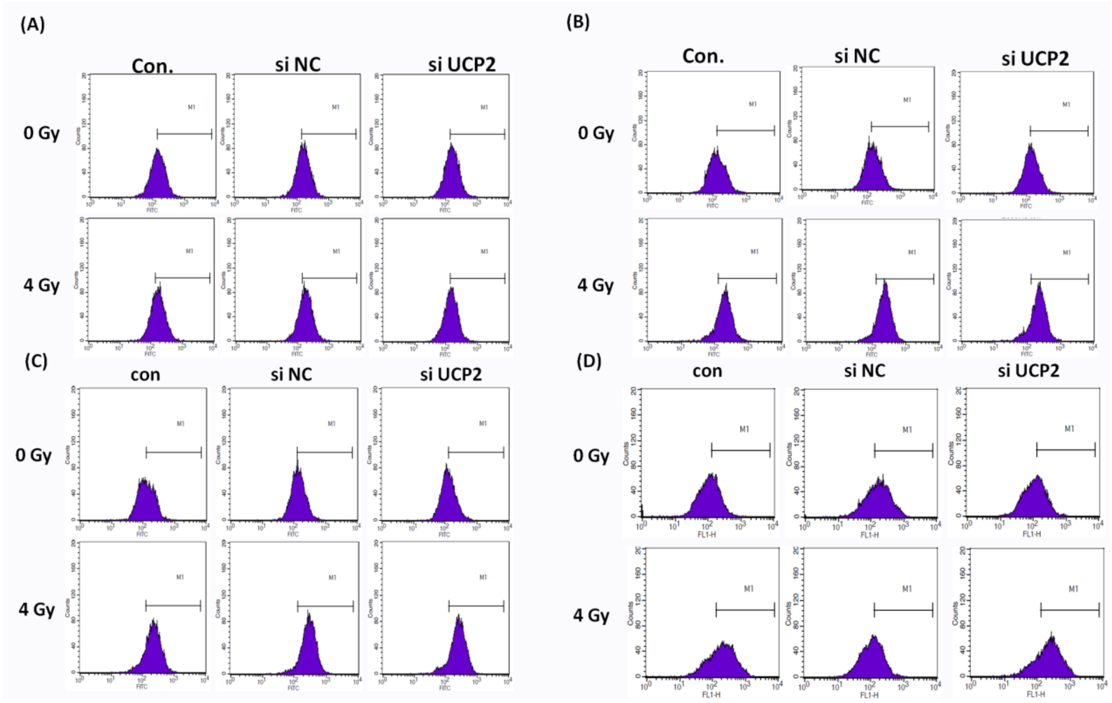

S1. Flow diagram of Alteration of mitochondrial membrane potential

(A,B,C,D)Alteration of mitochondrial membrane potential after irradiation in 6h,8h,12h,24h
